# Nanomaterials-Based Fluorimetric Methods for MicroRNAs Detection

**DOI:** 10.3390/ma8052809

**Published:** 2015-05-22

**Authors:** Ming La, Lin Liu, Bin-Bin Zhou

**Affiliations:** 1 College of Chemistry and Chemical Engineering, Pingdingshan University, Pingdingshan 467000, Henan, China, E-Mail: xrc1202@gmail.com; 2 College of Chemistry and Chemical Engineering, Anyang Normal University, Anyang 455000, Henan, China

**Keywords:** microRNAs, biomarker, fluorescence, metal nanomaterials, quantum dots, graphene oxide, silicon nanoparticles

## Abstract

MicroRNAs (miRNAs) are small endogenous non-coding RNAs of ~22 nucleotides that play important functions in the regulation of many biological processes, including cell proliferation, differentiation, and death. Since their expression has been in close association with the development of many diseases, recently, miRNAs have been regarded as clinically important biomarkers and drug discovery targets. However, because of the short length, high sequence similarity and low abundance of miRNAs *in vivo*, it is difficult to realize the sensitive and selective detection of miRNAs with conventional methods. In line with the rapid development of nanotechnology, nanomaterials have attracted great attention and have been intensively studied in biological analysis due to their unique chemical, physical and size properties. In particular, fluorimetric methodologies in combination with nanotechnology are especially rapid, sensitive and efficient. The aim of this review is to provide insight into nanomaterials-based fluorimetric methods for the detection of miRNAs, including metal nanomaterials, quantum dots (QDs), graphene oxide (GO) and silicon nanoparticles.

## 1. Introduction

MicroRNAs (miRNAs) are endogenous non-coding RNAs of ~22 nucleotides that regulate gene expression by translational repression or degradation of messenger RNA. The human genome may encode over 1000 miRNAs, which may target about 60% of mammalian genes and are abundant in many human cell types. Recently, miRNAs have been identified as diagnostic and prognostic biomarkers and predictors of drug response for many diseases, including a broad range of cancers, heart disease, and neurological diseases [1,2,3]. Since miRNAs are small molecular, high sequence homologous, and in low abundance in cancer cells (as low as a few molecules per cell), high sensitivity and specificity of a miRNAs detection system are very important for diagnosis and therapy of the cellular disease [4,5]. At present, Northern blotting technology, microarray and real-time PCR are being widely used for miRNAs analysis. However, these methods possess tedious sample preparation or require a thermal cycler. Recently, the simple and signal-amplified methods with the aid of nanomaterials have attracted great attention in bioassays. Nanomaterials contributed their high sensitivity, long life, and large surface area to the fabrication of different kinds of optical and electrochemical miRNAs biosensors [6]. Moreover, nanopore-based nucleotide analysis is an emerging technique that obviates the use of labeling, enzymatic reaction or amplification [7,8,9,10]. The studies in detecting cancer-derived miRNAs with nanopore implied that, in the next few years, the nanopore-based miRNAs technique may be validated for noninvasive and early diagnosis of diseases with the improvement of throughput. Progress in the fabrication of nanopore- and nanomaterials-based electronic miRNAs biosensors have been reviewed recently [6,11,12]. Fluorimetric methodologies in combination with nanotechnology are especially rapid, sensitive and efficient. Usually, nanomaterials can be used as the fluorophores or quenchers and as carriers for loading a large amount of probes to enhance the detection signal in the fluorescent sensing analysis. The recent advances in the development of nanomaterials-based fluorescent methods for miRNAs detection are summarized in this work, including metal nanomaterials, quantum dots (QDs), graphene oxide (GO), and silicon nanoparticles.

## 2. Metal Nanomaterials

### 2.1. Silver Nanoclusters

As promising alternatives to common fluorophores like organic dyes, few-atom metal nanoparticles (NPs) with strong and robust fluorescence emission have recently attracted considerable research interest. In particular, the creation of fluorescent silver nanoclusters (AgNCs) as new, bright, and photostable labels has received significant attention in the area of bioassays. When significantly small (less than 100 atoms) to exclude continuous density of states, discrete transitions between energy levels are possible for emission to occur. In order to achieve the creation of these small silver clusters and to avoid aggregation into larger nonemissive particles, a myriad of different scaffolds have been used. Typically, cytosine-rich DNA-templated fluorescent AgNCs (DNA/AgNCs) show great potential as fluorescent probes for biochemical applications due to their advantages of ultrafine size, low toxicity, good biocompatibility, outstanding photophysical properties, as well as facile integration with nucleic acid-based target-recognition abilities and signal amplification mechanisms [13,14,15,16]. Herein, we first summarized the development of AgNCs-based fluorescent probe design and its successful applications in detecting miRNAs. 

**Figure 1:**
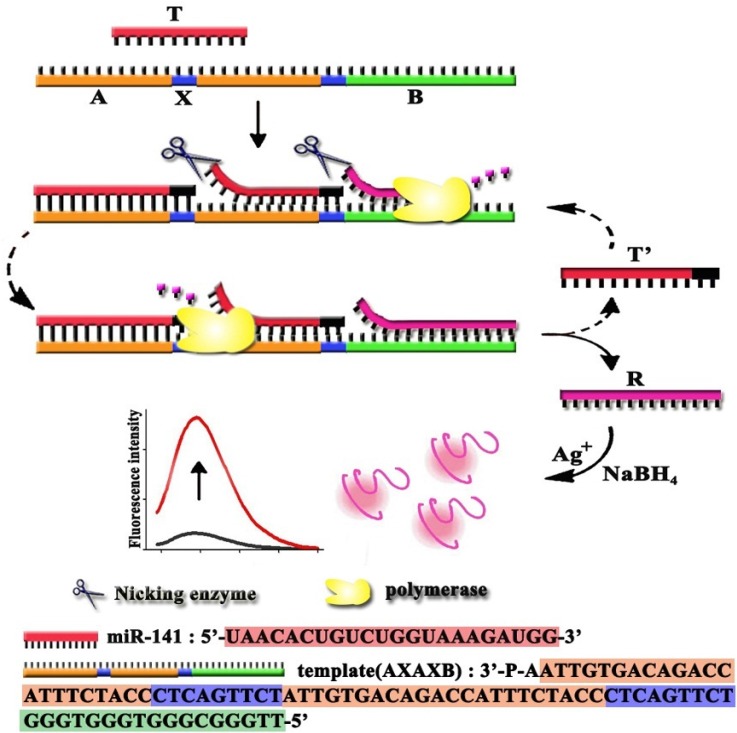
Detection of miRNA with attomolar sensitivity based on target-assisted isothermal exponential amplification (TAIEA) coupled with fluorescent DNA scaffolded AgNC probe. Reprinted with permission from [21]. Copyright 2012 American Chemical Society.

Based on the fluorescence properties of DNA/AgNCs, Yang’s group synthesized a DNA/AgNCs probe that can detect the presence of target miRNAs and investigated the effect of a range of diverse salts, organic solvents and buffer on the analytical performance [17,18,19]. The red fluorescence of the DNA/AgNC probe is diminished upon the presence of target miRNAs without pre- or post-modification, addition of extra enhancer molecules, and labeling. When target miRNAs are present, the emission of the DNA-nanosilver clusters (DNA/AgNCs) probe is significantly lower *versus* the case when no target miRNAs or other miRNAs are present. Additionally, to further advance the method toward multiplex miRNAs detection in solution, they also presented the design of three DNA/AgNCs probes showing green, red, and near-infrared (NIR) fluorescence [20]. Besides, Ye and co-workers reported the quantification of miRNAs expression levels in cell lysates by target assisted isothermal exponential amplification (TAIEA) coupled with fluorescent DNA-scaffolded AgNCs [16,21]. As shown in Figure 1, the unimolecular template involves five regions (AXAXB). Two repeat sequences of A are complementary to the target miRNAs. Also, two repeat sequences of X represent the “heart” of the template, upon their replication; the complementary strand includes the specific sequence for nicking by Nt·BstNBI. The sequence of B is complementary to the reporter oligonucleotide R acting as a scaffold for the synthesis of fluorescent AgNCs. After the amplification, the reporter oligonucleotide R was acting as a scaffold for the synthesis of fluorescent silver nanoclusters in the presence of Ag^+^ through the reduction of NaBH_4_.

**Figure 2:**
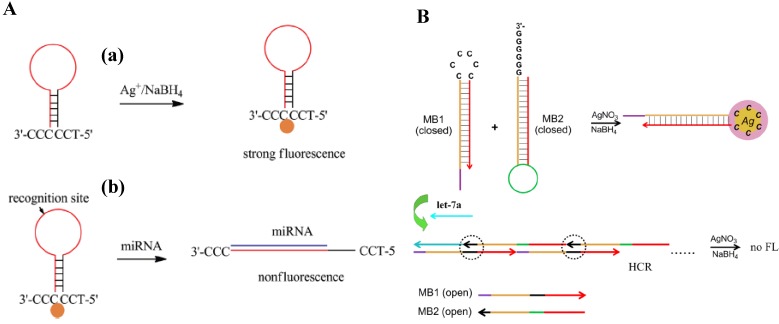
(**A**) Schematic representation of the creation of hairpin-shaped ODN probe/AgNCs for miRNA assay. (a) Template synthesis of AgNCs with strong fluorescence. (b) Hairpin-shaped ODN probe/AgNCs exhibited nonfluorescence or weak fluorescence after hybridization with target miRNA. Reprinted with permission from [22]. Copyright 2014 Elsevier. (**B**) Schematic illustration of HCR modulated fluorescent DNA-hosted Ag nanoclusters for the detection of the let-7a. Reprinted with permission from [24]. Copyright 2014 Elsevier.

Molecular beacon (MB) composing of a hairpin-like DNA stem-loop structure has been widely used for nucleic acid detection with excellent sensitivity and selectivity. Usually, the hairpin DNA probe was coupled with a fluorophore-quencher pair at its 5′- and 3′-termini. Upon hybridization with the target, the MB will undergo a spontaneous conformational reorganization, forcing the stem apart and causing the fluorophore and the quencher to separate from each other. As a result, the restoration of fluorescence occurs for detection assay. Recently, a few MB-based molecular detection platforms have been developed for miRNAs detection by using AgNCs as the fluorophores [22,23,24]. For example, Xia *et al.* found that the short single-stranded oligonucleotide probe with only six bases (5′-TCCCCC-3′) can serve as the scaffold for the preparation of DNA/AgNCs. On the basis of this finding, a hairpin DNA probe with 5′-TCC/CCC-3′ overhangs has been utilized for miRNAs assay. As shown in Figure 2A, the overhangs of 5′-TCC/CCC-3′, which were bought into close proximity by intramolecular self-hybridization, served as the template for the synthesis of fluorescent AgNCs (a). Upon hybridization with target miRNAs (b), the hairpin-shaped oligodeoxynucleotide (ODN) was destroyed, resulting in the separation of the 5′-TCC/CCC-3′ overhangs and the quenching of the AgNCs fluorescence. Moreover, Qiu *et al.* reported a DNA/AgNCs-based miRNAs detection platform based on hybridization chain reaction (HCR) using two complementary nucleic acid hairpin oligmers (MB1 and MB2) (Figure 2B) [24]. At first, MB1 and MB2 were in the closed form and spatially separated without the target miRNAs (let-7a). After the addition of AgNO_3_ followed by the reduction with NaBH_4_, highly fluorescent AgNCs were formed with the 6C loop in MB1 as the template. However, upon hybridization with let-7a, the hairpin structure of MB1 was opened, exposing a new single-stranded region that induced the opening of hairpin structured MB2, and the exposing of a new single strand of MB2 (identical in sequence to let-7a). This process led to the formation of a nicked double helix (polymer). Thus, the 6C-loop of MB1 was completely hybridized with the 6G sticky end of MB2, inhibiting the formation of fluorescent AgNCs.

### 2.2. Copper Nanoclusters

**Figure 3:**
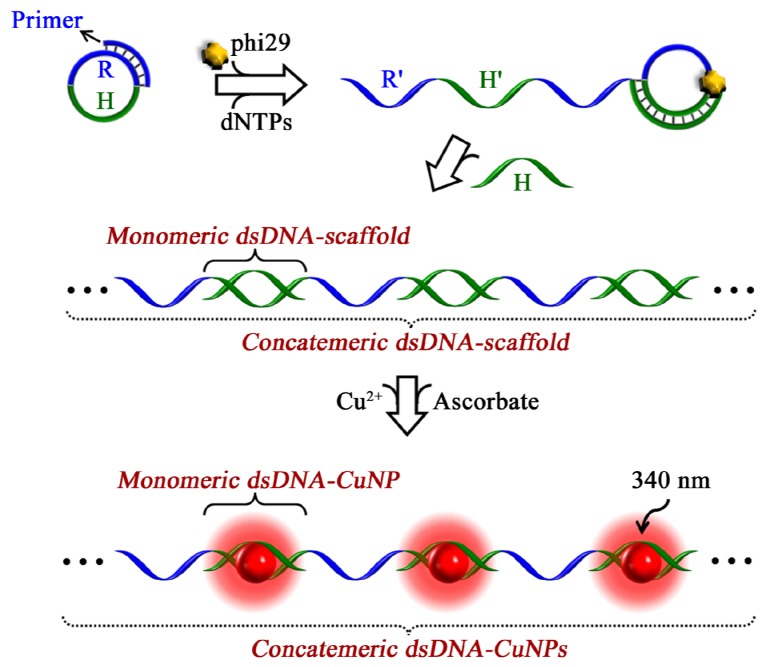
Schematic showing the principle of the RCR-mediated concatemeric dsDNA-CuNPs strategy. Reprinted with permission from [29]. Copyright 2014 American Chemical Society.

Compared with the other existing fluorescent metal nanoparticles, copper nanoclusters (CuNCs) or copper nanoparticles (CuNPs) are a type of newly emerged functional biochemical fluorescent probe [25,26,27]. In contrast to DNA/AgNCs, the newly emerging CuNCs, selectively formed on a DNA duplex, offer excellent potential for “on the spot” testing with a rapid and simple “mix-and-measure” format. For example, dsDNA-templated CuNCs (dsDNA-CuNCs) can be facilely prepared by reducing Cu^2+^ ions with ascorbic acid within fifteen minutes and the Cu^2+^ ions are soluble in many detection environments and thus have no precipitation phenomena like the Ag^+^ ions. For these views, Wang *et al.* presented a label-free method for miRNAs detection using fluorescent dsDNA-CuNCs as signal indicators [28]. In this method, the miRNAs targets were transferred to the oligonucleotide reporters and acted as the scaffold for the synthesis of fluorescent CuNCs via an isothermal exponential amplification reaction, in which the unimolecular DNA designed for the miRNAs target is used as the amplification template and polymerases and nicking enzymes were used as mechanical activators [28]. Furthermore, Xu *et al.* reported the detection of miRNAs using concatemeric dsDNA-templated CuNPs (dsDNA-CuNPs) by introducing the rolling circle replication (RCR) technique into CuNPs synthesis [29]. In this strategy, the circular DNA template contained two functional regions (R and H) (Figure 3). A part of the recognition region R is complementary to the primer. The hybridization region H was designated to template the formation of CuNPs in its dsDNA form. In the presence of phi29 polymerase and dNTPs, the primer triggered the RCR process with a continual replication of the circular template. As a result, a short oligonucleotide primer was extended to a long concatemeric ssDNA with periodically repeated complementary parts of regions R and H (named as R′ and H′). Through ensuing hybridization of the H′ region with complementary DNA H, a long concatemeric dsDNA scaffold comprising two distinct alternating regions of R′ ssDNA and H/H′ dsDNA was obtained to synthesize concatemeric dsDNA-CuNPs after the addition of copper ions and ascorbic acid. In comparison with monomeric dsDNA-CuNPs, the sensitivity of concatemeric dsDNA-CuNPs was greatly improved with ~10,000-fold amplification.

### 2.3. Gold Nanopartilces

**Figure 4:**
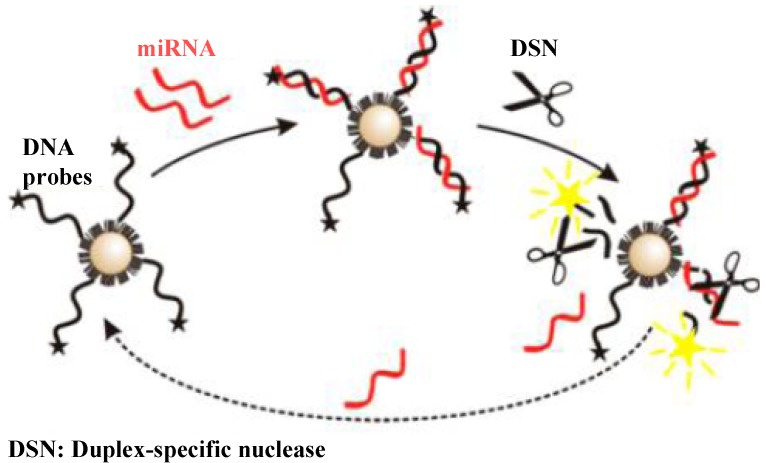
Assay strategy. Reprinted with permission from [34]. Copyright 2014 American Chemical Society.

The attractive characteristics of gold nanoparticles (AuNPs) such as high surface-to-volume ratio, high extinction coefficients, unique size-dependent optical properties and good conductance properties have proven to be of high utility in biomedical applications [12,30]. Recently, AuNPs-based fluorescent assays for miRNAs detection have been reported based on the ultraefficient fluorescence quenching efficiency of AuNPs [31,32]. For example, Tu *et al.* has demonstrated the detection of miRNAs by coupling AuNPs distance-dependent fluorescence quenching with a conformation-switched hairpin-structured probe that was labeled with thiol and a fluorophore [33]. The hairpin-structured probe DNA was anchored onto AuNPs surface via the formation of the Au–S bond. The stem-loop feature of the probe brings the fluorophore close to the AuNP surface to silence the fluorescence and no detectable fluorescence signal was observed. The miRNAs targets opened the loop of the probe by hybridization, leading to the fluorophore to be away from the AuNPs surface, the follow-up recovery of the fluorescence and the production of a readily detectable signal. The detection limit of this method was found to be 0.01 pM. Furthermore, Degliangeli *et al.* introduced a fluorescent method for the absolute quantification of miRNAs based on enzymatic processing of DNA-functionalized AuNPs [34]. Specifically, fluorescently labeled DNA probes were immobilized on the passivation layer of PEGylated AuNPs, inducing the efficient fluorescence quenching by the vicinity of the fluorophores to the AuNPs surface (Figure 4). In presence of target miRNAs, DNA-RNA heteroduplexes were formed, followed by hydrolyzation by the duplex-specific nuclease (DSN) enzyme. As a result, fluorophores were released in solution, leading to the appearance of a fluorescence signal. Note that the DSN is a highly stable, nonspecific endonuclease with can selectively cleave double stranded DNA and DNA in DNA-RNA heteroduplexes with little activity toward single-stranded nucleic acids and RNA-RNA duplex helixes [35]. Thus, miRNAs strands remained intact in this process and would be released back to the sample solution for recycling, making signal enhanced significantly. The detection limit was found to be 5~8 pM (or 0.2~0.3 fmol) after 5 h incubation.

Based on the fluorescence quenching efficiency of AuNPs, Baptista’s group suggested that AuNPs functionalized with a fluorophore labeled hairpin-DNA (Au-nanobeacon) can be used to follow the synthesis and inhibition of RNA [36]. Furthermore, they proposed an innovative Au-nanobeacon-based theranostic approach for the detection and inhibition of sequence-specific miRNA *in vitro* [37]. The proposed method allows real-time detection of the beacon's signal while yielding a quantifiable fluorescence directly proportional to the level of gene silencing. 

## 3. Quantum Dots

**Figure 5:**
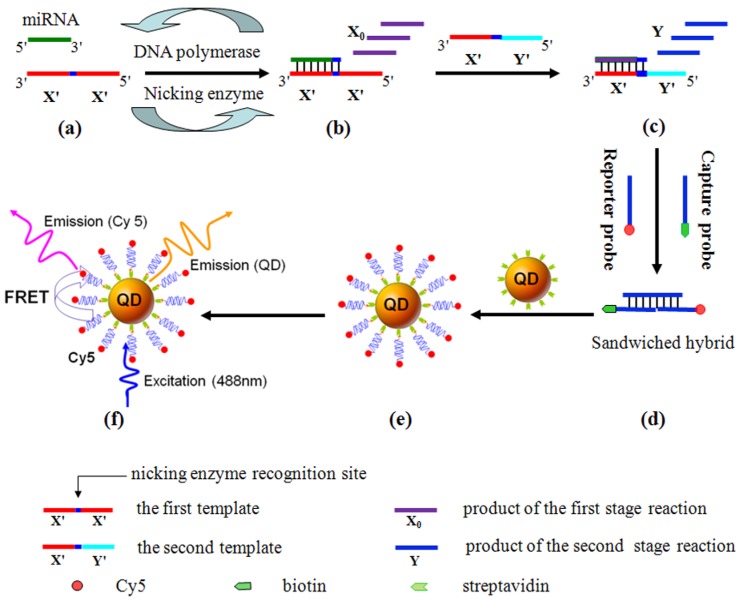
Scheme of the miRNA assay based on the two-stage EXPAR and single-QD-based nanosensor. Reprinted with permission from [44]. Copyright 2012 American Chemical Society.

**Figure 6:**
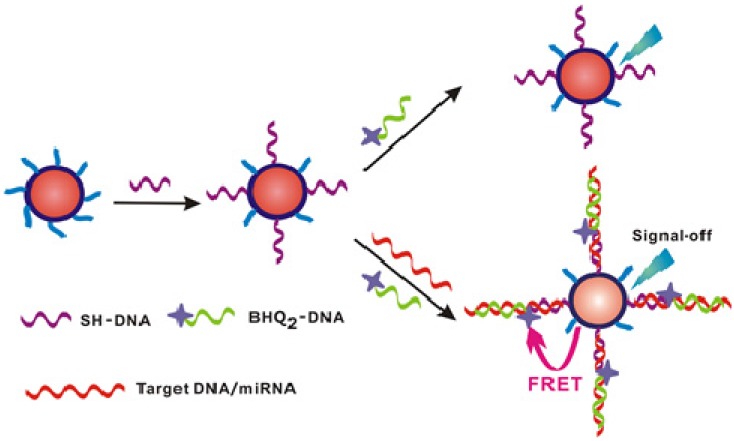
Schematic representation of the designed nanosensors for detection of DNA/miRNA based on the FRET system. Reprinted with permission from [45]. Copyright 2012 American Chemical Society.

Quantum dots (QDs) are novel semiconductor nanocrystals with unique optical properties, including size-tunable photoluminescence spectra and relatively high quantum yield, and have been widely used as fluorescent markers in biological labeling, fluorescence imaging, and drug delivery [38,39,40,41]. In particular, QDs hold great promise as fluorescence resonance energy transfer (FRET) donors in the biosensing applications to homogeneously detect nucleic acids, proteins, and small molecules [42]. The use of QDs as the FRET donors offers several unique spectroscopic properties unmatched in any available organic fluorophores, including improved FRET efficiency as a result of coupling multiple acceptors around a single QD, tunable spectral overlap between the QD and the acceptor, minimization of direct acceptor excitation, and multiplex FRET configurations [43]. Recently, the fluorescent detection techniques in combination with QDs have been used to quantify miRNAs with high sensitivity [44,45]. Typically, Zeng *et al.* developed a QD-based miRNAs nanosensor for a point mutation assay using primer generation-mediated rolling circle amplification (PG-RCA) [46]. Zhang *et al.* reported a miRNAs detection method based on the two-stage exponential amplification reaction (EXPAR) and a single-QD-based nanosensor [44]. As shown in Figure 5, the two-stage EXPAR involves two templates and two-stage amplification reactions under isothermal conditions. The first-stage reaction (a, b) is an exponential amplification with the involvement of the X′-X′ template, which can enable the amplification of miRNAs. The second-stage reaction (c) is a linear amplification with the involvement of the X′-Y′ template, which can enable the conversion of miRNAs to the reporter oligonucleotide Y. It should be noted that different miRNAs can be converted to the same reporter oligonucleotides, which can be detected with the same set of capture and reporter probes without the need for resynthesis of the specific DNA probes for each new target miRNAs or reoptimization of the assay conditions. After amplification, the reporter oligonucleotide Y is sandwiched by a biotinylated capture probe and a Cy5-labeled reporter probe (d). This sandwich hybrid is then assembled on the surface of a 605QD to form the 605QD/reporter oligonucleotide Y/Cy5 complex through specific biotin-streptavidin binding (e). When this complex was excited by a 488 nm argon laser, the fluorescence signals of 605QD and Cy5 is observed simultaneously due to FRET from 605QD to Cy5 (f). Additionally, Su *et al.* reported a versatile “signal-off” strategy for miRNAs detection using CdTe/CdS core-shell QDs capped with 3-mercaptopropionic acid (MPA) (Figure 6) [45]. Surface-bound short-chain MPA molecules were first substituted by thiolated DNA via ligand exchange, leading to programmable DNA modification at the surface of QDs. The resulting DNA-conjugated QDs were used as fluorescence nanoprobes for miRNAs detection with strong photoluminescence and robust stability. Without the addition of a target miRNAs sequence, the organic quencher (BHQ_2_)-labeled DNA had almost no influence on the DNA-QDs conjugate. With the addition of target miRNAs and BHQ2-labeled DNA sequences, sandwiched hybrids were formed. As a result, the fluorescence intensity of DNA-QDs decreased obviously with the addition of target miRNAs due to the energy transfer from QDs to BHQ_2_. Moreover, Jou *et al.* reported a two-step miRNAs detection platform with semiconductor CdSe/ZnS QDs modified by FRET quencher-functionalized DNA. Specifically, the DNA sequence on the QDs surface included the recognition sequences for target miRNAs and telomerase primer sequences for the second step of the analytical platform. In the presence of DSN, subjecting the DNA probe-modified QDs to target miRNAs induced the formation of miRNA/DNA duplex helixes. The follow-up DSN-mediated cleavage could lead to the regeneration of target miRNAs. The DSN-induced cleavage of the quencher units resulted in the activation of the fluorescence of the QDs, thus allowing for the fluorescent detection of miRNAs (the first step). The DNA residues associated with the QDs after cleavage of the DNA probe by DSN acted as primers for telomerase. The subsequent telomerase/dNTPs stimulated elongation of the primer units formed G-quadruplex telomer chains. Incorporation of hemin in the resulting G-quadruplex telomer chains yields horseradish peroxidase-mimicking DNAzyme units, catalyzing the generation of chemiluminescence in the presence of luminol/H_2_O_2_ (the second step) [47].

**Figure 7:**
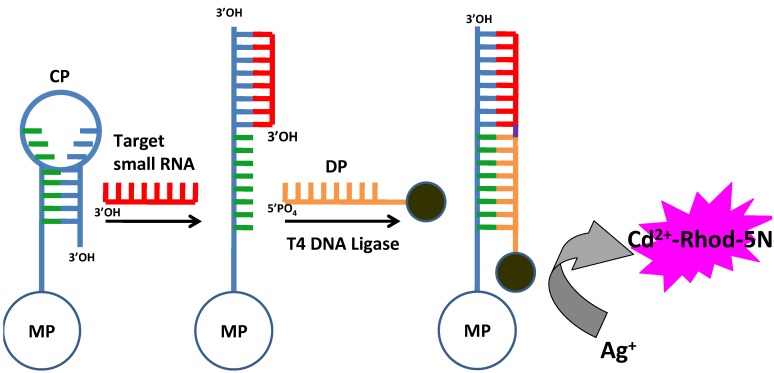
Schematic presentation of the small RNA detection assay using CXFluoAmp. MP: magnetic particles; CP: capture probe; DP: detection probe. Reprinted with permission from [49]. Copyright 2009 American Chemical Society.

It has been suggested that each CdSe nanocrystal with an average diameter of 5.15 nm contains 2051 Cd^2^^+^ ions and every 1 nm increase in nanocrystal diameter represents 2200 more Cd^2^^+^ ions enclosed [48]. The large numbers of encapsulated ions can be released by a fast and gentle process, the cation exchange reaction. For this consideration, Li *et al.* reported the quantification of miRNAs in biological samples based on the signal amplification of CdSe QDs [49]. As shown in Figure 7, the capture probes are conjugated onto the magnetic beads and the detection probes are coupled with the nonfluorescent CdSe nanocrystals. The capture probe with a molecule beacon structure opened up and exposed the binding site for the detection strand upon hybridization of target miRNAs. Then, the nanocrystals were immobilized onto the magnetic beads. Afterwards Cd^2^^+^ ions were released to react with the Rhod-5N dye to produce intense fluorescent signals.

## 4. Graphene Oxide

**Figure 8:**
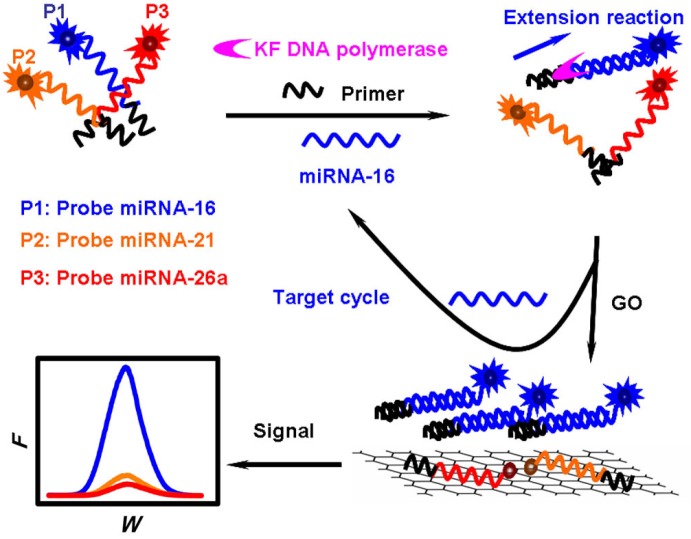
Illustration of the GO fluorescence quenching and ISDPR-based multiple miRNA analysis. Reprinted with permission from [58]. Copyright 2012 American Chemical Society.

Graphene oxide (GO), a single-atom-thick, two-dimensional carbon nanomaterial, has become extremely popular in biological applications due to its unique characteristics, such as good water-solubility, flexible modification and super fluorescence quenching ability. In particular, it has been proven that GO can adsorb single-stranded nucleic acids via non-covalent π–π stacking interactions between the hexagonal cells of graphene and the ring structure of nucleobases, but has less affinity toward rigid double-stranded nucleic acids [50,51]. GO has been used as a platform for the detection of nucleic acids because of its remarkable properties [52,53] and proteins [54,55]. It has also been suggested that GO sheets could bind dye-labeled single-stranded DNA probes and efficiently quench the fluorescence of the labeled fluorescent-single-strand DNA. In the presence of target miRNAs, the labeled probes can be released from GO due to the formation of miRNA/DNA duplex helixes that disturb the interaction between the labeled DNA probes and GO. Then, the fluorescence can be recovered. This simple method could be used for assay of miRNAs at the nanomolar level [56,57]. However, the miRNAs content is at the attomolar to femtomolar level in many biological samples. Thus, great efforts have been made to develop more sensitive GO-based fluorescent sensors for the detection of low-abundance miRNAs with different signal amplification strategies [58]. Typically, Dong *et al.* presented a simple, highly sensitive, and selective multiple miRNAs detection method based on the GO fluorescence quenching and isothermal strand-displacement polymerase reaction (ISDPR) [58]. As shown in Figure 8, the high fluorescent quenching efficiency of GO by a FRET-based mechanism in combination with strong interaction between ssDNA and GO made the probes labeled with fluorescent dye exhibit minimal background fluorescence. Upon the recognition of specific target miRNAs, an ISDPR was triggered to produce numerous massive specific miRNA/DNA duplex helixes, and a strong emission was observed due to the weak interaction between the DNA-miRNA duplex helix and GO. The large planar surface of GO made it possible to simultaneously quench several DNA probes with different dyes and obtain a multiple biosensing platform for the detection of different target miRNAs in the same solution.

**Figure 9:**
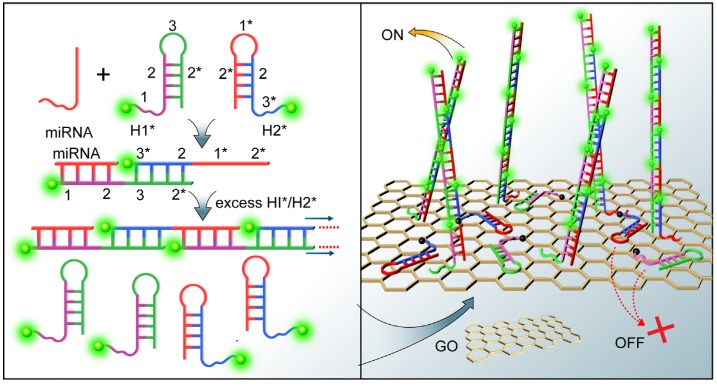
Schematic illustration of the proposed HCR/GO platform for miRNA detection. Reprinted with permission from [59]. Copyright 2012 American Chemical Society.

Hybridization chain reaction (HCR) is enzyme-free amplification method that has shown great potential in nucleic acid detection. Yang *et al.* developed an enzyme-free signal amplified method for miRNAs detection using HCR coupled with a GO surface-anchored fluorescence signal readout pathway (Figure 9) [59]. In this method, miRNAs initiated HCR between two species of fluorescent hairpin probes in solution. When GO was added into the solutions after HCR, both of the excess hairpin probes and the HCR products were anchored onto the GO surface via the π–π stacking interaction. The fluorescence of the hairpin probes was completely quenched by GO, whereas the HCR products maintained strong fluorescence. 

**Figure 10:**
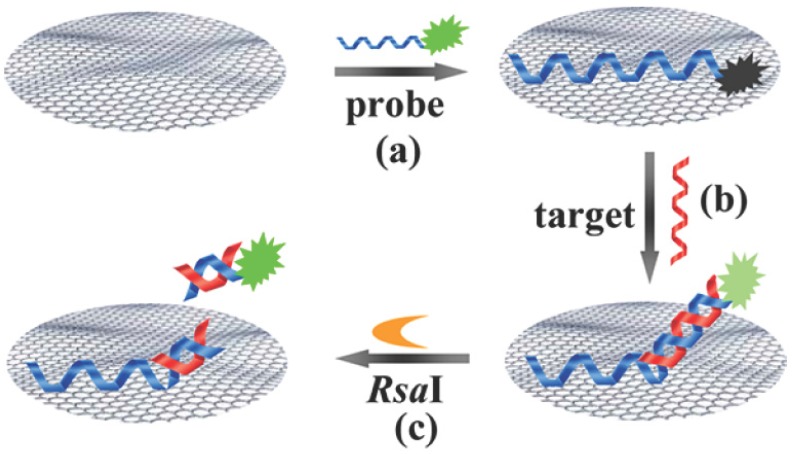
Illustration of miRNA assay based on coupling the fluorescence quenching of GO with site-specific cleavage of an endonuclease. The designed P1, which is labeled at its 5′-terminus with a fluorophore, exhibits partial complementarity to the target (T1). The adsorption of P1 on the GO surface effectively quenches the fluorophore (step a). The hybridization of P1 with T1 leads to a non-GO absorbed duplex region and a single-stranded domain that is associated with the GO surface (step b). In the presence of the endonuclease, the 5′-terminus of the duplex (containing the RsaI-recognized tetranucleotide sequence 5′-GTAC-3′) is digested, resulting in the release of the fluorophore to the solution and recovery of the fluorescence signal (step c). The recovered fluorescence signal depends on the target concentration in solution. Reprinted with permission from [60]. Copyright 2013 American Chemical Society.

RsaI endonuclease is a site-specific endonuclease that recognizes the duplex symmetrical tetranucleotide sequence 5′-GTAC-3′ and catalyzing cleavage between T and A bases. Tu *et al.* reported the miRNAs assay by coupling the fluorescence quenching efficiency of GO with site-specific cleavage of RsaI endonuclease for improving selectivity (Figure 10) [60]. The designed single-stranded probe DNA carries both a binding region (44 bases) and a sensing region (22 bases). The binding region provides an anchoring function to facilitate the interaction between GO and the probe, inducing fluorescence quenching of the 5′-terminus-labeled fluorophore (6-carboxyfluorescein, FAM). The sensing region specifically recognizes the target miRNAs and hybridizes with it to form a duplex, which contains the specific sequence recognized by RsaI endonuclease. In the absence of a specific target, no fluorescence signal was detected. In the presence of target miRNAs, however, the formed duplex was subject to be released from the GO surface under the cleavage of RsaI endonuclease, resulting in the recovery of fluorescence of the fluorophore and producing a readily detectable signal. Moreover, Guo *et al.* reported a fluorescent sensing platform for miRNAs detection by combining the fluorescence quenching efficiency of GO and DSN-induced target recycling [61]. In the absence of target miRNAs, fluorophore-labeled DNA probes would be adsorbed by GO, leading to fluorescence quenching. In the presence of target miRNAs, the DSN cleaved the labeled DNA in the DNA-RNA hybrid duplex into small fragments and the miRNAs was released from the duplex for recycling, producing numerous small fluorophore-labeled DNA fragments that could not adsorb onto the GO surface. 

**Figure 11:**
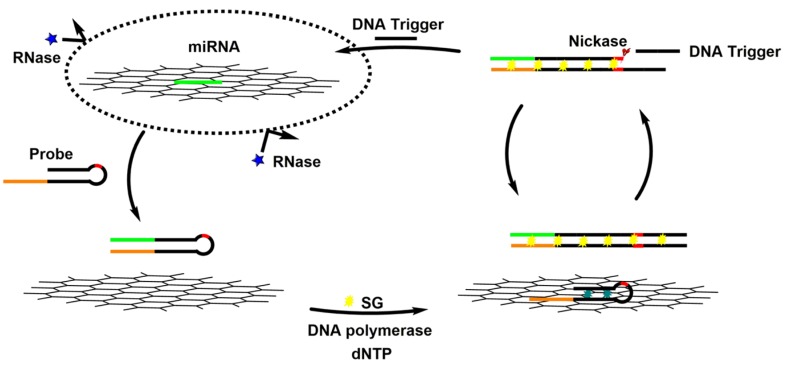
Illustration of the Graphene Fluorescence Switch-Based Cooperative Amplification for Target miRNAs. Reprinted with permission from [64]. Copyright 2014 American Chemical Society.

It has been demonstrated that ssDNA and single-stranded RNA (ssRNA) probes were effectively protected from enzymatic digestion by nuclease after noncovalent adsorption onto GO surface due to a steric hindrance effect of GO that prevents nuclease from binding to the DNA and RNA [62]. Besides combining the extraordinary fluorescence quenching property of GO with the unique ssDNA/GO interaction to elaborately design miRNAs sensors in homogeneous solution, researchers have also been inspired to develop new types of GO-based miRNAs sensors. For example, DNase I is an endonuclease that nonspecifically cleaves DNA (single- and double-stranded DNA, chromatin and DNA stranded in RNA/DNA complex) but not RNA and releases di-, tri- and oligonucleotide products; Cui *et al.* reported a signal amplification platform for multiplex analysis of miRNAs by combining DNase I and a single-labeled DNA fluorescent probe adsorbed on GO [63]. Specifically, single-stranded DNA was promptly adsorbed onto GO forming strong molecular interactions that prevented DNase I from approaching the constrained ssDNA. However, when hybridized with the target miRNAs, the double-stranded miRNA/DNA bound weakly to GO and released into solution, where the DNA probe could immediately be hydrolyzed by DNase I, while the miRNAs remained intact. The released miRNAs could then bind to another probe on GO to initiate a next round of cleavage. Moreover, Liu *et al.* developed a stable, sensitive, and specific miRNAs detection method on the basis of cooperative amplification combining with the GO fluorescence switch-based circular exponential amplification (CEA) and the multimolecules labeling of SYBR Green I (SG) [64]. As shown in Figure 11, the target miRNAs adsorbed on the surface of GO protected it from enzyme digest. If the miRNAs hybridized with a partial hairpin probe acting as a primer to initiate a strand displacement reaction to form a complete duplex, universal DNA fragments would be released under the action of nicking enzyme and used as triggers to initiate next reaction cycle, constituting a new circular exponential amplification. In the proposed strategy, a small amount of target miRNAs can be converted to a large number of stable DNA triggers, leading to a remarkable amplification for the target. Moreover, compared with labeling in a 1:1 stoichiometric ratio, multimolecules binding of intercalating dye SG to double-stranded DNA (dsDNA) can enhance the fluorescence signal significantly, improving the detection sensitivity. 

**Figure 12:**
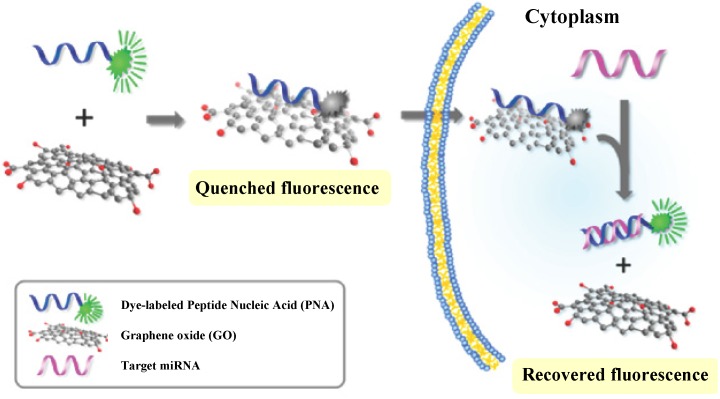
Scheme of strategy for a miRNA sensor based on NGO and PNA. Reprinted with permission from [67]. Copyright 2013 American Chemical Society.

Although GO has been utilized not only for the detection of miRNAs *in vitro* as a fluorescence quencher of dye-labeled DNA probes but also for the delivery of nucleic acid integrated molecular beacon with help of cationic polymers [65,66], quantitative monitoring of intracellular miRNAs in living cells still remains an important challenge. Recently, Ryoo *et al.* developed a nanosized GO (NCO)-based sensor for quantitative monitoring of target miRNAs expression levels in living cells (Figure 12) [67]. The binding of NCO with fluorophore-labeled peptide nucleic acid (PNA) probe induced fluorescence quenching. The presence of target miRNAs led to the recovery of the fluorescence PNA. In this work, PNA acting as a probe for miRNAs sensing offers many advantages such as high sequence specificity, high loading capacity on the NCO surface compared to DNA and resistance against nuclease-mediated degradation. The sensor allowed the detection of specific target miRNAs with the detection limit as low as ~1 pM and the simultaneous monitoring of three different miRNAs in a living cell.

## 5. Silicon

**Figure 13:**
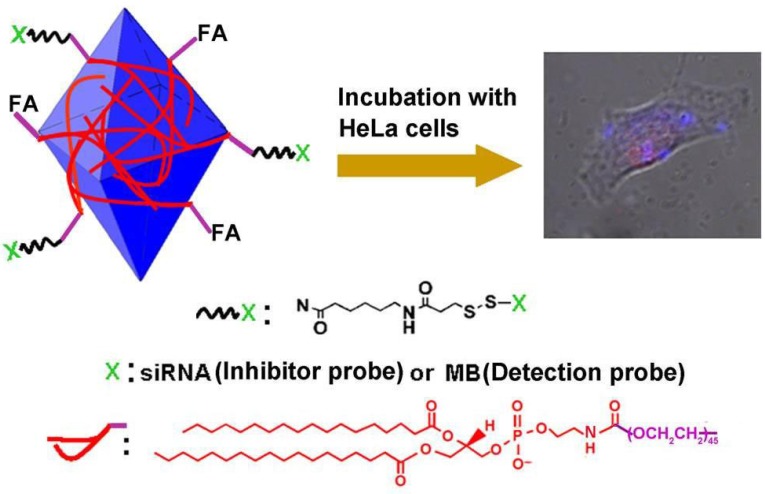
Schematic representation of mf-SnO2 nanoprobe for target-specific-cell imaging and intracellular detection of miRNA. FA = folic acid, MB = molecular beacon. Reprinted with permission from [73]. Copyright 2012 John Wiley and Sons.

Recent significant progress in optical imaging techniques has offered the opportunities of noninvasive and repeated real-time analysis of miRNAs in living cells [68]. Heavy metal inorganic nanoparticles have shown demonstrated use in spherical nucleic acid systems. However, the potential toxicity and biodegradability issues of metal nanoparticles remain a concern [4,69,70]. Silicon is well-established to be biocompatible, biodegradable, and earth-abundant, and can exhibit photoluminescence [71]. Recently, multifunctional silicon-based nanomaterials have been used for imaging intracellular target miRNAs. For example, Zhang *et al.* reported the detection of miRNAs in the cells positive to lung cancer using fluorescent silicon-based nanoshells as a molecular imaging agent [72]. These nanoshells were composed of silica spheres with encapsulated tris(2′, 2′-bipyridyl)dichlororuthenium(II) hexahydrate (Ru(bpy)_3_^2^^+^) complexes as cores and thin silver layers as shells. Dong *et al.* designed a multifunctional SnO_2_ nanoprobe (mf-SnO_2_) that contains a cell-targeting moity (folic acid, FA) for cell-specific delivery and a conjugated gene probe (molecular beacon, MB) for imaging intracellular target miRNAs (Figure 13) [73]. In this method, MB to detect target miRNAs is conjugated by a disulfide linkage, which is sensitive to pH values. Cleavage of the disulfide linkage between the gene probe and the nanoparticle enhances the efficiency of intracellular delivery. The stable aqueous suspension of SnO_2_ NPs were obtained by noncovalently functionalizing the SnO_2_ NPs with 1,2-distearoyl-*sn*-glycero-3-phosphoethanolamine-N-[amino(polyethylene glycol)2000] (DL-PEG_2000_) conjugated to FA (DL-PEG_2000_-FA) or sulfosuccinimidyl-6-(3′ (2-pyridyldithio) propionamido)hexanoate (DL-PEG_2000_-SPDP). These moieties were adsorbed on the nanoparticles by van der Waals and hydrophobic interactions.

**Figure 14:**
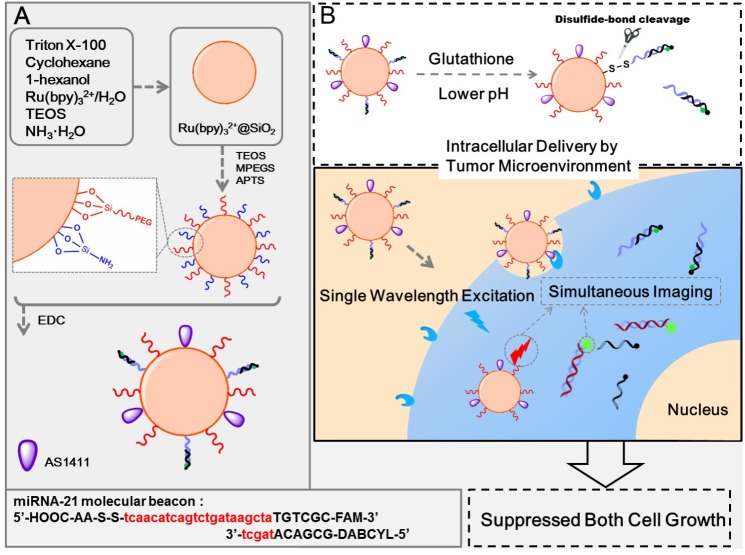
Schematic of the synthesis of FS-AS/MB and strategy of cancer-targeting theranostics using FS-AS/MB. Reprinted with permission from [74]. Copyright 2014 American Chemical Society.

Multicolor fluorescent bioimaging by single-wavelength excitation has been proved to be a powerful tool for simultaneous monitoring of multiple targets in cells. Recently, Li *et al.* designed a multifunctional target-cell-specific fluorescence SiO_2_ nanoprobe for target-cell-specific delivery, cancer cells and intracellular miRNAs imaging, and cancer cell growth inhibition (Figure 14) [74]. The nanoprobe (FS-AS/MB) was prepared by simultaneously coupling of the AS1411 aptamer and miRNA-21 molecular beacon (miR-21-MB) onto the surface of Ru(bpy)_3_^2+^-encapsulated silica (FS) nanoparticles. In the work, the FS nanoparticles were synthesized by a one-pot two-step reverse microemulsion method; Polyethylene glycol (PEG) and functionalized amine groups were conjugated to these FS nanoparticles, providing better biocompatibility and allowing subsequent bioconjugation, respectively. Cell-specific delivery was achieved by functionalizing FS nanoparticles with AS1411 aptamer, leading to the delivery of MB only inside targeted cells with nucleolin protein. The simultaneous cell and intracellular miRNAs imaging can be accomplished under the same excitation wavelength without any cross-talk. Most importantly, the released miR-21-MB from the nanoprobe can hybridize with miRNA-21 and inhibit cell growth *in vitro*.

## 6. Conclusions

MiRNAs, playing important functions in a number of developmental and physiological processes, have been regarded as promising biomarkers and therapeutic targets in cancer treatment. Nanomaterials-based sensing strategies offer numerous advantages over traditional molecular diagnostic, such as signal amplification, improved sensitivity and simplicity, as well as versatile sensing scheme that can be tailored to a desired target. Recently, considerable efforts have been made to enhance the sensitivity for miRNAs detection by utilizing the unique chemical and physical properties of nanostructures. This work reviewed the progress in the development of fluorimetric methodologies for miRNAs detection based on functional nanoscaffolds of novel nanomaterials, such as metal nanostructures, QDs, GO and SnO_2_ nanoparticles. Their analytical performances were shown in Table 1. Although there are still limitations for their practical use as regular methods in clinical diagnostic and prognostic, the advances in nanoscience and nanotechnology promise a better future for the sensing industries. 

**Table 1:** Comparison of the performances of nanomaterials-based fluorimetric miRNAs biosensors.

Nanomaterials	Signal Amplification	Detection ranges	Detection limits	References
AgNCs	-	0~1.5 μM	<0.25 μM	[18]
AgNCs	-	5~125 nM	1.7 nM	[22]
AgNCs	HCR	1.56~400 nM	0.78 nM	[24]
AgNCs	Target recycling	0.5~50 nM	0.16 nM	[23]
AgNCs	TAIEA	10 aM~1 nM	2 aM	[21]
CuNCs	RCR	10~400 nM	10 pM	[29]
CuNCs	TAIEA	1 pM~10 nM	1 pM	[28]
AuNPs	-	0.05~50 pM	0.01 pM	[33]
AuNPs	DSN	-	<25 pM	[34]
CdSe/ZnS	DSN	0.53 pM~3.9 nM	0.28 pM	[47]
CdTe/CdS	-	10 fM~10 nM	10 fM	[45]
CdSe nanocrystals	Cation-Exchange	0.1 pM~5 μM	35 fM	[49]
488QDs	PG-RCA	0.1 fM~1 nM	50.9 aM	[46]
605QDs	EXPAR	0.1 aM–10 fM	0.1 aM	[44]
GO	-	50~400 nM	-	[56]
GO	Endonuclease	20 pM~1 nM	9 pM	[63]
GO	HCR	1 pM~5 nM	-	[59]
GO	DSN	0.5 pM~1 nM	160 fM	[61]
GO	CEA	0.06~12 pM	10.8 fM	[64]
GO	Endonuclease	0.02~100 pM	3 fM	[60]
GO	ISDPR	5 fM~5 pM	2.1 fM	[58]
Nano GO	-	0~100 nM	2 nM	[57]
Nano GO	-	0~1 μM	1 pM	[67]
SiO_2_	-	0.5~40 nM	0.18 nM	[74]
